# Albumin-Coated Single-Core Iron Oxide Nanoparticles for Enhanced Molecular Magnetic Imaging (MRI/MPI)

**DOI:** 10.3390/ijms22126235

**Published:** 2021-06-09

**Authors:** Abdulkader Baki, Amani Remmo, Norbert Löwa, Frank Wiekhorst, Regina Bleul

**Affiliations:** 1 Fraunhofer Institute for Microengineering and Microsystems IMM, Carl-Zeiss-Straße 18-20, 55129 Mainz, Germany; Abdulkader.Baki@imm-extern.fraunhofer.de; 2 Physikalisch-Technische Bundesanstalt, Abbestraße 2-12, 10587 Berlin, Germany; Amani.Remmo@ptb.de (A.R.); Norbert.Loewa@ptb.de (N.L.); Frank.Wiekhorst@ptb.de (F.W.)

**Keywords:** iron oxide nanoparticles, serum albumin, magnetic particle spectroscopy, magnetic particle imaging, magnetic resonance imaging

## Abstract

Colloidal stability of magnetic iron oxide nanoparticles (MNP) in physiological environments is crucial for their (bio)medical application. MNP are potential contrast agents for different imaging modalities such as magnetic resonance imaging (MRI) and magnetic particle imaging (MPI). Applied as a hybrid method (MRI/MPI), these are valuable tools for molecular imaging. Continuously synthesized and in-situ stabilized single-core MNP were further modified by albumin coating. Synthesizing and coating of MNP were carried out in aqueous media without using any organic solvent in a simple procedure. The additional steric stabilization with the biocompatible protein, namely bovine serum albumin (BSA), led to potential contrast agents suitable for multimodal (MRI/MPI) imaging. The colloidal stability of BSA-coated MNP was investigated in different sodium chloride concentrations (50 to 150 mM) in short- and long-term incubation (from two hours to one week) using physiochemical characterization techniques such as transmission electron microscopy (TEM) for core size and differential centrifugal sedimentation (DCS) for hydrodynamic size. Magnetic characterization such as magnetic particle spectroscopy (MPS) and nuclear magnetic resonance (NMR) measurements confirmed the successful surface modification as well as exceptional colloidal stability of the relatively large single-core MNP. For comparison, two commercially available MNP systems were investigated, MNP-clusters, the former liver contrast agent (Resovist), and single-core MNP (SHP-30) manufactured by thermal decomposition. The tailored core size, colloidal stability in a physiological environment, and magnetic performance of our MNP indicate their ability to be used as molecular magnetic contrast agents for MPI and MRI.

## 1. Introduction

Molecular imaging provides an integrative technology to study biological processes using non-invasive visualization of specific molecules in vivo [1] with the aim of early disease diagnosis and treatment evaluation [2]. To this end, numerous imaging techniques have been developed for molecular imaging such as magnetic resonance imaging (MRI) [1], positron emission tomography (PET) [3], computed tomography (CT) [4], single-photon emission computed tomography (SPECT) [5,6], or ultrasound (US) [7]. Aside from the physical principle (detection of frequency, radionuclides, emitted light, X-ray, etc.), these modalities are different regarding invasiveness and cost-effectiveness.

MRI relies on detecting the nuclear magnetic resonance signal of protons of the hydrogen atom ^1^H after applying radiofrequency pulses. Therefore, MRI is not specific to molecules and requires the use of contrast agents or tracers to provide specificity and sensitivity for molecular imaging. Due to their magnetic properties, contrast agents such as paramagnetic Gd-compounds can shorten the *T*_1_ (or longitudinal) and *T*_2_ (or transverse) relaxation time of neighboring water protons. These effects increase the signal intensity of *T*_1_-weighted images (positive contrast, contrast-agent-containing regions appear brighter) or reduce the signal intensity of *T*_2_-weighted images (negative contrast, region with contrast agent appears darker). Transition metal ions, such as high-spin manganese (II) and iron oxide nanoparticles (iron-(III) oxides), are known to strongly affect the *T*_2_ relaxation and represent the typical content of negative contrast agents [8,9].

Generally, magnetic nanoparticles (MNP) constitute a class of potent contrast agents providing positive or negative contrast depending on their concentration and on the MRI sequences used [10]. To characterize an MRI contrast agent, the relaxivities *r*_1_ and *r*_2_ are considered, that is, the ability of the contrast agent (normalized to the iron concentration *c*(Fe)) to increase the longitudinal *R*_1_ = 1/*T*_1_ and transversal *R*_2_ = 1/*T*_2_ relaxation rate of the proton magnetization. By immobilization or aggregation of the MNP, e.g., by binding to biomolecular targets or after internalization into cells, these contrast properties might be drastically changed [11]. Even though iron-oxide-based MNP were already clinically approved in the late 90 s as negative MRI contrast agents for liver MRI (Feridex, Endorem, Resovist) [12], most of them were withdrawn from the European market in the meantime due to claims such as limitation of their field applications, deficiency of diagnostic impact, and economic factors. Additionally, the occurrence of harmful side effects was reported caused either by labile iron or hypersensitivity reactions attributed to the coating of the MNP [13,14]. However, ferumoxytol (Feraheme or Rienso) is approved for iron-replacement therapies, and additional approval is being sought for imaging applications, which are beyond its authorized indication. To date, iron-oxide-based MNP have attracted growing attention as a newly discovered biocompatible alternative to the clinically widely used gadolinium(Gd)-based contrast agents [15]. For instance, (ultra)small iron oxide nanoparticles are currently investigated as potential MRI positive contrast agents to replace the critically observed Gd agents due to their potentially harmful side effects [16,17,18,19].

A further imaging modality with high potential in sensitive molecular imaging is magnetic particles imaging (MPI), first presented in 2005 [20]. MPI stands out due to high spatial (below mm) and excellent temporal (1–10 ms) resolution without any ionizing radiation exposure [20]. Though MPI so far is not in clinical use for medical applications, its high potential for diagnostic vascular or perfusion imaging, imaging-guided vascular interventions [21], or cancer diagnostics is acknowledged [20,22]. MPI has reached preclinical levels, but still requires the development of suitable MNP as tracers with improved dynamic magnetic and biological properties such as sufficient magnetic response, blood half-life time, and stability in a physiological environment.

Since MPI is a direct imaging technique that specifically detects the MNP, the generated MP images are background-free, and subsequently, no anatomical information is provided. However, combining the high resolution 3D anatomical MRI data with molecular tracking of MNP tracers by MPI constitutes a promising hybrid imaging technology [23,24]. Besides their application as tracers in diagnostic imaging, MNP can also be utilized as functional components in therapeutic applications for, e.g., hyperthermia and drug delivery [25,26,27].

Depending on their intended application, MNP require specific properties for an optimal performance in these different fields. For instance, according to theoretical models and preliminary experimental studies of different groups, single-core iron-oxide MNP with a distinct size of about 25–30 nm diameter are indicated to be effective tracers for MPI [25,28]. Moreover, investigations on magnetosomes (single-core iron-oxide MNP grown by biotechnological processing) have already demonstrated a good performance for MPI as well as for magnetic hyperthermia [29,30,31]. The provision of single-core iron oxide MNP in this size range requires highly controllable synthesis strategies. Conventional batch synthesis approaches such as thermal decomposition, microemulsion, the sol–gel method, ultrasonication, and coprecipitation are either time-consuming and require phase transfer or display considerable batch-to-batch variations in both size and size distribution, leading to undefined magnetic properties [32]. A more robust synthesis method is the continuous micromixing approach, which has already been shown to provide good control of particle size and magnetic characteristics in aqueous synthesis [32,33]. Here, the stages of nucleation and particle growth can be isolated as a function of distance. The position where initial mixing induces nucleation is spatially separated from the particle growth occurring in the ripening zone. Due to in situ stabilization during this microfluidic production process, electrostatically stable dispersed single-core MNP in an aqueous medium are obtained. As described in the literature, alternative approaches, e.g., laser target evaporation from bulk material, can also produce stable aqueous magnetic nanofluids even without the need for an electrostatic stabilizer [34]. Coating with a double layer of oleic acid of magnetic nanoparticles obtained by combustion synthesis was reported as a biocompatible strategy for self-stabilizing particle dispersions in water [35]. Stable aqueous nanoparticle dispersions provide a safe starting product for biomedical development. Further surface modification can be performed straightforwardly by coating without preceding phase transfer. MNP surface characteristics play a key role in signal generation in medical imaging, as they influence the dispersion stability in the physiological environment and act as a linker for further functionalization. Moreover, the surface coating of iron oxide MNP is reported to improve biocompatibility and reduce cytotoxic effects that can be caused by reactive oxygen species (ROS) [36,37,38]. Potent agents for molecular imaging are supposed to prevent non-specific interaction with the biological system, e.g., with serum proteins, high ionic strength, blood and endothelial cells, while specifically targeting biological markers or receptors. The attachment of specific ligands helps to identify signal molecules or bind to and detect, e.g., cancer cells that overexpress typical receptors.

The biocompatibility, non-toxicity, and stability of MNP are crucial for in vivo applications. The MNP surface can be modified with different strategies utilizing macromolecules with different functional groups such as dextran [39,40], polyethylene glycol (PEG) [41,42], or numerous peptides [43]. Serum albumin is a main blood plasma protein with significant vital functions such as maintaining the pH and osmotic pressure of blood and exhibit excellent stability in physiological systems [44,45]. Moreover, the functional groups existing in albumin such as amino and carboxy groups enable it to bind different drug-targeting ligands. For instance, Abraxane^®^, an albumin–paclitaxel nanoparticle, is a clinically approved chemotherapeutic drug conjugate. Its efficacy was confirmed in clinical trials and showed a significant activity in pancreatic cancer patients [46] and antitumoral effects in women with breast cancer [47]. Mesken et al. have investigated plasmid-loaded albumin nanoparticles for cellular uptake and gene delivery [48]. Due to its similar biological function and chemical composition, bovine serum albumin (BSA) as analogue to human serum albumin (HSA) is a widely used component for investigations and developments in the field of drug delivery and cancer diagnostic [49]. Many research groups have also used BSA for MNP surface coating after synthesis, mainly resulting in clusters of small MNP in a size range between 4 and 10 nm [50,51,52,53]. Kalidasan et al. have investigated the conjugation of BSA to 10 and 30 nm non-spherical, rhombus-shaped MNP using cetyl trimethyl ammonium bromice (CTAB) and 3-Amino-Trimethoxysilane (APTM) as linker molecules. Due to the improved colloidal stability of these BSA-MNP, the specific absorption rate (SAR) values for magnetic hyperthermia could be increased from 1700 W/g for MNP without BSA to 2300 W/g for BSA-MNP [54]. To conclude, for a successful surface modification, not only does the coating material have to be carefully selected, but also effects occurring due to the required post-synthesis procedures such as phase transfer or purification steps might lead to clustering effects or undesirable residue of solvents that can be disadvantageous for their application as contrast agents.

In this study, we demonstrate that surface modification of continuously synthesized single-core MNP with BSA effectively improves their stability and ensures a robust performance as tracers in molecular imaging. Manufacturing and surface modification were carried out in an aqueous medium without the addition of harmful chemicals or the need for phase transfer. The as-synthesized (tannin-coated) and BSA-coated MNP were comprehensively studied regarding changes in their colloidal stability and magnetic properties at different physiological relevant salt (NaCl) concentrations. Average size, size distribution, and particle morphology were investigated by transmission electron microscopy (TEM) and differential centrifugal sedimentation (DCS). Changes in particle surface were studied by Zeta potential measurements and gel electrophoresis. Using AC-susceptibility (ACS) measurements of the linear magnetic susceptibility, changes in the hydrodynamic properties of the MNP systems have been investigated. To determine the MPI performance of the MNP, we used magnetic particle spectroscopy (MPS). MPS detects the non-linear dynamic magnetic response of MNP exposed to an alternating magnetic field. Since it is based on the same physical mechanisms, it can be considered zero-dimensional MPI. Furthermore, to evaluate the capability of the MNP systems as contrast agents in MRI, nuclear magnetic resonance (NMR) relaxivity measurements were carried out. For comparison, MPS and NMR measurements of the two commercial MNP systems Resovist (multi-core system, MRI liver contrast agent) and SHP-30 (single-core nanoparticle system synthesized by thermal decomposition, preclinical research) have been included.

## 2. Results and Discussion

The capability of a continuous micromixer technique to synthesize aqueous single-core MNP with diameters of up to 40 nm has been demonstrated recently [32]. By adjusting the two synthesis parameters *T*_S_ = 55 °C and residence time *t*_R_ = 4 min, MNP with mean core size diameter of about *d*_c_ = 30 nm were found to possess high performance in MRI and MPI.

Based on this MNP system, further improvement using surface modification with a BSA protein coating is envisaged regarding stability in a physiological environment and magnetic performance for molecular imaging applications. We show that centrifugation significantly improves the magnetic performance of the as-synthesized MNP in Section 2.1, demonstrate the structural and magnetic changes by surface modification using a BSA-coating in Section 2.2, and present the steady stability of the BSA-coated system in different physiological saline concentrations together with the maintenance of the powerful MRI and MPI imaging capability of this MNP system. For comparison, all measurements were performed with as-synthesized MNP (tannic acid coated) without any further surface modification and the centrifuged MNP as well. Furthermore, we included data of the two commercial systems Resovist and SHP-30.

### 2.1. Particle Fractionation by Centrifugation

**Figure 1:**
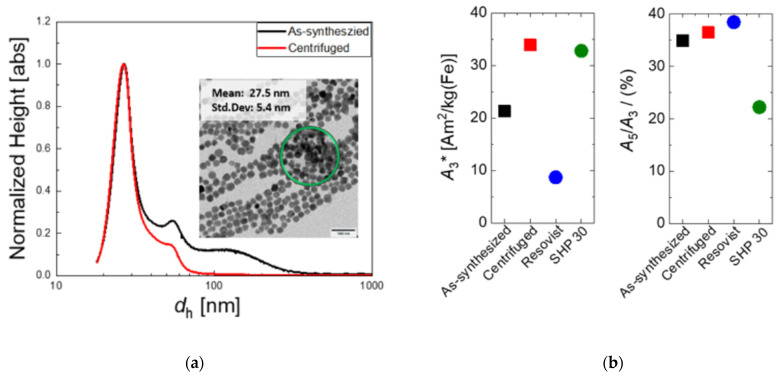
(**a**) Particle fractionation by centrifugation (prior to BSA-coating): Hydrodynamic size distribution measured by DCS of as-synthesized (black line) and centrifuged (red line) MNP, where the broad shoulder from 70 up to 300 nm is significantly reduced. Inset: Representative TEM image of as-synthesized MNP with areas of aggregated particles marked by the green circle (scale bar 100 nm). (**b**) MPS parameters A_3_* and *A_5_/A_3_* measured at *B*_ex_ = 25 mT for as-synthesized (black symbols) and centrifuged MNP (red symbols) showing the signal increase gained by particle fractionation. Included are the corresponding MPS parameters of the two commercial systems Resovist (blue symbols) and SHP-30 (green symbols).

Our continuous micromixer synthesis approach provides stable dispersions of single-core nanoparticles with narrow size distribution, as shown in Figure 1a. Spherical single-core particles with a mean diameter *d*_c_ of 27.6 ± 5.5 nm were determined by TEM analysis of the as-synthesized MNP (containing the basic tannic acid coating). The apparent hydrodynamic diameter *d*_h_ distribution determined by DCS shows a narrow peak with a maximum at 26.5 nm close to, but slightly smaller than, the core diameter obtained by TEM. Notably, the hydrodynamic size distribution obtained by DCS for the as-synthesized and centrifuged MNP shown in Figure 1a assumes a density of pure magnetite neglecting the decrease in density resulting from the organic tannic acid coating on the surface. This leads to slightly shifting the hydrodynamic diameters determined by DCS to smaller values (as the real density of the particles is smaller than the assumed magnetite density used for the data analysis).

Furthermore, a minor fraction with *d*_h_ > 30 nm of MNP attributed to agglomerates or superstructures is observed in DCS. These larger entities might affect the signal generation in magnetic imaging due to undesired particle–particle interaction of the particle magnetic moments. Therefore, we performed an additional centrifugation prior to BSA-coating and analyzed the resulting structural and magnetic changes of the particle fractionation.

**Figure 2:**
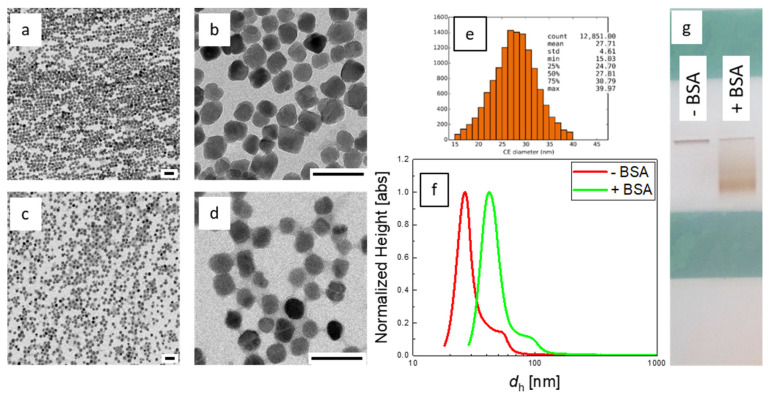
Physicochemical characterization of continuously synthesized MNP. TEM images (scale bar 100 nm) of centrifuged MNP without BSA-coating (**a**,**b**) and with BSA-coating (**c**,**d**). Core diameter histogram from TEM image analysis of uncoated MNP (**e**). Hydrodynamic size distribution obtained by DCS (**f**) of centrifuged MNP without BSA (red line), and with BSA-coating (green line). Agarose gel electrophoresis (**g**) shows a high mobility of BSA-coated MNP (+BSA, **right**) and nearly immobilized MNP without BSA-coating (−BSA, **left**).

DCS measurements of the centrifuged MNP system confirm the successful separation of agglomerates with *d*_h_ > 80 nm, as illustrated in Figure 1a. The centrifugation results in a yield reduction of about 35%, as estimated from the corresponding MNP content of the individual fractions determined by iron concentration analysis. The small shoulder right to the main peak in DCS still indicates the tendency to form small superstructures such as dimers or trimers, which in our experience is reversible. The DCS results are confirmed by TEM, where no clustering is found in TEM images of the MNP system after centrifugation, as shown in the next section (see Figure 2a).

The effect of centrifugation is also expressed in the magnetic properties of the MNP. As displayed in Figure 1b, the MPS measurements showed an increase of about 37% of the specific (MPS moment normalized to iron content) A_3_* amplitude from 21.3 Am^2^/kg(Fe) to 33.9 Am^2^/kg(Fe) by centrifugation. This indicates the remarkable enhancement of signal sensitivity as a consequence of isolating stable single-core MNPs from the fraction-containing agglomerates. The MNP concentration-independent shape parameter *A_5_/A_3_* showed a slight increase from 34.9% to 36.4%. Generally, this increase of the *A*_5_/*A*_3_ parameter results in a better MPI performance and image resolution.

The centrifuged MNP system was then used for surface modification by BSA-coating, as described in the next section.

### 2.2. BSA-Coating of Single-Core Magnetic Nanoparticles for Imaging Applications

Generally, iron oxide nanoparticles are stabilized in situ during continuous micromixer synthesis with tannic acid, which acts mainly as an electrostatic stabilizing agent. However, high salt concentrations as present in a physiological environment (typically 150 mM NaCl, isotonic saline) strongly affect the colloidal stability of electrostatically stabilized nanoparticles. Thus, a further modification, preferably with a steric stabilizer, is required. In biomedical and pharmaceutical applications, biocompatible synthetic as well as biologic polymers are commonly used, e.g., polyethylene glycol and copolymers, polypeptides or proteins, and polysaccharides [55,56].

In this study, bovine serum albumin (BSA) was chosen for surface modification to prevent aggregation of the single-core magnetic nanoparticles. This blood plasma protein has high concentrations in blood and is known to form a protein corona to particles injected in the blood stream very rapidly [57]. BSA-coating is a frequently reported strategy [50] to prevent non-specific interaction and enhance colloidal stability and biocompatibility of nanoformulations. Here, we evaluated the effect of BSA-coating as further surface modification on the structural and magnetic imaging properties and compared them with the corresponding behavior of the uncoated systems.

TEM analysis of the original, centrifuged sample compared to the BSA-treated sample confirmed the successful protein coating (Figure 2a–d). The size and shape of both samples were relatively homogenous and displayed mostly spherical single-core particles. No significant changes of the cores were observed after the coating procedure. The mean core size of centrifuged sample was estimated by automatic image analysis and showed 27.7 nm with relative standard deviation of 0.17 (Figure 2e). Though the presence of protein coating is poorly visible in TEM analysis due to the low contrast of BSA, the effect of the coating increasing the interparticle distances is clearly seen in the images (Figure 2a–d) indicating the successful coating. An increase of the apparent hydrodynamic diameter from *d*_h_ = 26.5 nm to *d*_h_ = 42 nm after BSA-coating was determined by DCS analysis (Figure 2f), where the effective density of magnetite and a BSA monolayer was taken into account this time (see methods section for details). From the *d*_h_ difference, a mean BSA layer thickness of about 8 nm could be estimated.

Changes in the surface characteristics of the MNP after BSA-coating were investigated by zeta potential measurements and agarose gel electrophoresis. The latter technique is commonly used for the separation and analysis of biomacromolecules, e.g., oligonucleotides. Separation depends generally on the (surface) charge as well as the molecular weight. It has been shown that gel electrophoresis is also a useful tool to analyze surface charge, size, and stability of nanoparticles [58]. The as-synthesized MNP possess a high negative surface charge with a zeta potential of about −51 mV. This highly negative value can be explained by anionic polyphenolic groups of the tannic acid coating. The BSA-coated sample displayed a value of about −25 mV in distilled water, which agrees with values for BSA at pH 6.5 in the literature [34]. Generally, sufficient electrostatic stabilization of nanoparticles can be assumed for samples with a zeta potential that exceeds 20 mV, irrespective of their sign [59]. Apparently, the MNP without BSA-coating did not pass the electrophoresis gel, as shown in Figure 2g. This was attributed to the high affinity of tannic acid to polysaccharide that might have caused crosslinking and hydrogel formation, as reported in the literature [60]. In contrast, BSA-coated samples displayed significant migrations through the gel, as displayed in Figure 2g. This qualitative finding evidences that BSA-coating was successful and changed the surface characteristics of the MNP.

**Figure 3:**
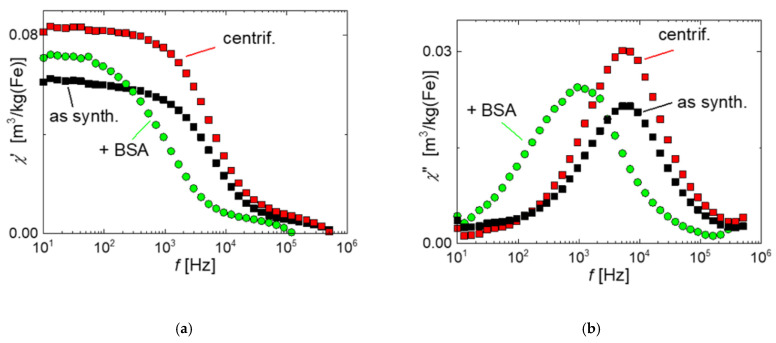
Linear AC susceptibility versus frequency of as-synthesized (black symbols), centrifuged (red symbols), and BSA-coated (green symbols) MNP. (**a**) real part *χ*′, (**b**) imaginary part *χ*′′. While as-synthesized and centrifuged MNP show the same spectra with an increased susceptibility for the centrifuged MNP of about 26%, the BSA-coated MNP show a shift of the *χ*′ peak towards lower frequencies due to the increase of hydrodynamic diameter by the additional BSA-layer.

The effect of BSA-coating on the magnetic properties of the MNP was investigated by ACS and MPS measurements. ACS measurements in Figure 3 display the typical shape observed for a colloidal MNP system with a step-like decrease of the real part *χ*′ with increasing frequency (Figure 3a) and a distinct maximum in the imaginary part *χ*′′(*f*) of the complex linear susceptibility (Figure 3b). The as-synthesized and centrifuged MNP show nearly identical spectra, only differing in the linear susceptibility amplitude *χ*_0_, which increases by about 26% from *χ*_0_ = 0.062 m³/kg(Fe) for the as-synthesized to 0.084 m³/kg(Fe) for the centrifuged MNP. This shows the improvement in the magnetic quality of the MNP without changing the fraction of sizes responsible for the dynamic magnetic behavior. In other words, the increase in amplitude is attributed to the effective removal of the aggregates by centrifugation, which, due to their inhibited Brownian rotation, do not contribute notably to the linear susceptibility in the measured frequency range. The *χ*′′ (*f*) curves of both uncoated systems have the maximum at nearly the same frequency of *f* = 5.92(2) kHz, indicating that by centrifugation the hydrodynamic size distribution of the free MNP is not changed in agreement with the DCS measurements after centrifugation (see Figure 1a).

The increase in hydrodynamic diameter by adding the BSA-layer was apparent in ACS measurements as a shift of the peak position of the imaginary part *χ*′′ from about 5 kHz to about 1 kHz (see Figure 3b). The increased hydrodynamic diameter of the BSA-coated particles augments the rotational inertia of the moments to follow the oscillating excitation field, resulting in the observed shift of the *χ*′′ peak towards lower frequencies. At the same time the susceptibility amplitude *χ*_0_ (*χ′* for *f*⟶0) is slightly decreased by about 14% after BSA-coating, still remaining above the amplitude of the as-synthesized MNP (Figure 3a). This indicates the effective coating of the MNP by the BSA layer.

**Figure 4:**
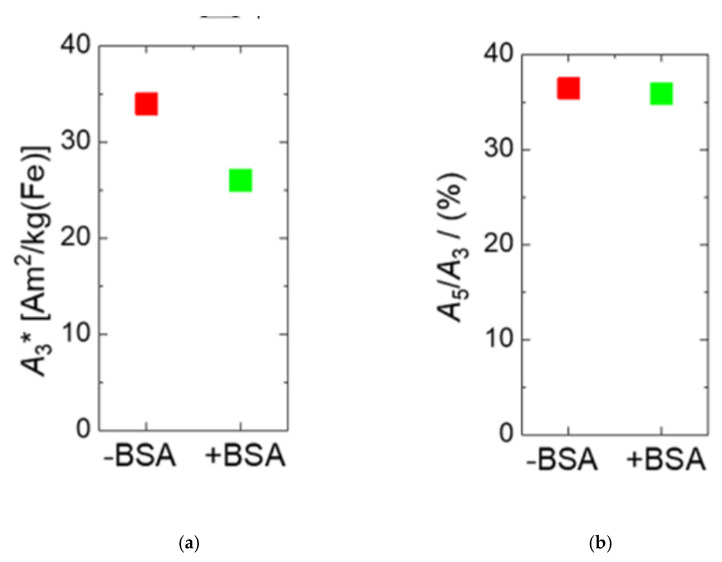
MPS parameters A_3_* (**a**) and *A*_5_*/A*_3_ (**b**) of uncoated MNP (red symbols) and BSA-coated MNP (green symbols). The specific MPS amplitude A_3_* is reduced by about 20% while the shape parameter *A*_5_/*A*_3_ remains nearly unchanged. This reduction is attributed to the increase in hydrodynamic diameter from 26.5 to 42 nm by BSA-coating.

As displayed in Figure 4a, the MPS specific amplitude decreased slightly from *A*_3_* = 33.9 Am^2^/kg(Fe) to 26 Am^2^/kg(Fe) after BSA-coating, which is still a remarkably high value that is three times higher than the value A_3_* = 8.7 Am^2^/kg(Fe) of Resovist. The reason for the *A*_3_* reduction is assumed to again be the increase of hydrodynamic diameter by the additional BSA-coating reducing to some extent the capability of the magnetic moments of the MNP to follow the excitation magnetic field, diminishing the resulting magnetic response in MPS. Thus, both the observed reduction of the linear magnetic AC susceptibility amplitude *χ*_0_ and the measured decrease in the MPS signal amplitude *A*_3_* are caused by the increase of hydrodynamic diameter after BSA-coating. As displayed in Figure 4b, the *A*_5_*/A*_3_ ratio remained nearly constant at the comparable high level of 35.8% for BSA-coated MNP (compared to 36.4% for MNP without BSA), indicating that the BSA-coating will maintain the MPI performance.

Similar effects of reduced MPS performance have been observed for MNP where the local viscosity has been increased, leading to the same increase of rotational inertia of MNP moments [61].

**Figure 5:**
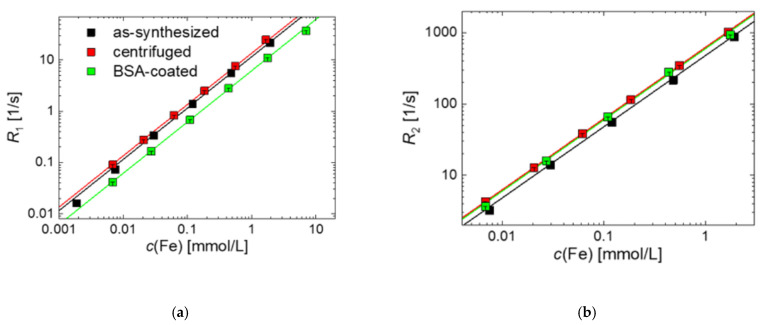
Room temperature NMR relaxation rates *R*_1_ (**a**) and *R*_2_ (**b**) measurements as a function of iron concentration *c*(Fe) of as-synthesized, centrifuged, and BSA-coated MNP measured at 1.5 T. The relaxation rates are obtained by linear regression and drawn as straight lines; the corresponding for the *r*_1_ and *r*_2_ values are listed in Table 1.

To assess the performance of BSA-coated MNP in the second magnetic imaging modality, MRI, we determined the NMR relaxivities *r*_1_ and *r*_2_, as shown in Figure 5.

The relaxation rates *R*_1_ = 1/*T*_1_ and *R*_2_ = 1/*T*_2_ as a function of iron concentration *c*(Fe) measured at *B*_0_ = 1.5 T are shown in Figure 5 for as-synthesized, centrifuged, and BSA-coated MNP. We obtained relaxivities of *r_1_* = 11.6(9) and *r*_2_ = 482(8) L mol^−1^ s^−1^, for as-synthesized MNP, *r_1_* = 13.6(6) and *r*_2_ = 620(7) L mol^−1^ s^−1^ for centrifuged MNP, and *r_1_* = 6.4(1) and *r*_2_ = 600(1) L mol^−1^ s^−1^ for BSA-coated MNP, indicating their high capability as negative contrast agents. For the MRI liver contrast agent Resovist, values of *r*_1_ = 7.4 and *r*_2_ = 95 L mol^−1^ s^−1^ are reported in literature at the same field *B*_0_ = 1.5 T [1,62]. The *r*_2_ relaxivity is about six times higher than Resovist. Additionally, the high ratio between transversal and longitudinal relaxivities *r_2_/r_1_* indicates the efficiency of a *T*_2_ as contrast agent. Hence, the coated MNP can be considered an alternative to Resovist with even better MR imaging performance.

Interestingly, we observe a higher relaxivity for BSA-coated particles, which at first argues against classical theory, since the diffusing hydrogen protons are shielded from the magnetic core. However, there is the possibility that the BSA shell prolongs the residence time of the hydrogen protons near the magnetic core, thereby leading to a higher relaxivity. Monte Carlo simulations contribute to the understanding of the relationship between layer thickness and changes in relaxivity [63].

To summarize, the comprehensive characterization of BSA-coated MNP indicates their competitive high performance in MRI and MPI (Table 1). Compared to commercial systems, our BSA-coated MNP surpass Resovist in both imaging modalities. The *r*_2_ relaxivity is about six times higher than Resovist. MPI signal amplitude *A*_3_* reaches that of SHP-30, a well-defined, commercial single-core MNP system manufactured by thermal decomposition and modified after phase transfer with an amphiphilic polymer.

### 2.3. Stability in Physiological Environment

Preserving the MNP stability in physiological systems is a key requirement for their in vivo clinical applicability [64]. Unprotected MNP exposed to serum proteins or isotonic salt concentrations will form aggregates and agglomerates with potential severe consequences for the biological system. Additionally, salt-induced agglomeration might affect the magnetic properties of MNP and thereby lower the diagnostic information of magnetic imaging modalities.

In this section, we incubate BSA-coated MNP as well as as-synthesized MNP (tannic acid coating) at three different saline concentrations, *c*(NaCl) = 0.05, 0.10, and 0.15 mol/L, to evaluate the stability of MNP after short- (2 h) as well as long-term (1 day, 1 week) incubation time. Colloidal stability changes impacting the hydrodynamic size distribution and aggregation were analyzed by DCS, while changes in magnetic properties were deducted from MPS measurements.

**Figure 6:**
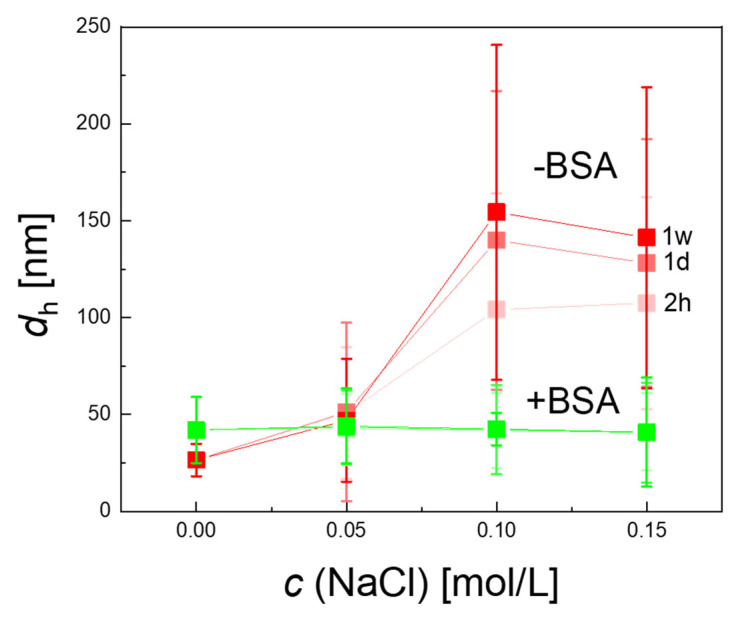
Colloidal stability of MNP. The hydrodynamic diameter as function of saline concentration (*c*(NaCl) = 0.05, 0.10, 0.15 Mol/L) measured by DCS 2 h (2 h), 1 day (1 d), and 1 week (1 w) after incubation. Notably, for BSA-coated MNP (green symbols), all three curves coincide within symbol thickness, proving that the hydrodynamic diameters *d*_h_ of BSA-coated MNP remain unchanged (<1%). For BSA-coated MNP (red symbols), *d*_h_ is already significantly increased at the lowest saline concentration. The full-width-at-half-maximum (FWHM) of the size distribution is displayed as uncertainty bars. The lines between the symbols are reading aids.

**Figure 7:**
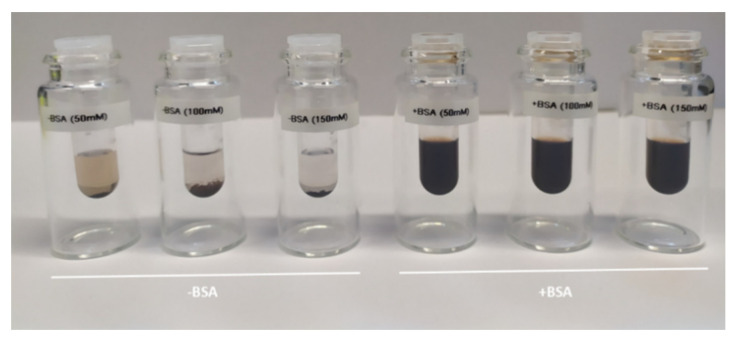
Visualization of colloidal stability of MNP at different saline concentrations. MNP without BSA-coating (left) and with BSA-coating. The photograph was taken one week after saline incubation at c (NaCl) = 0.05, 0.10, and 0.15 mol/L. While the uncoated MNP are almost entirely precipitated at the vial bottom, the BSA-coated MNP visually show a homogenous dispersion with no aggregation tendency.

The BSA-coated MNP remained highly stable over the complete observation time even at the highest saline concentration, c (NaCl) = 0.15 mol/L (Figure 6), without any noticeable aggregation visible in DCS. In contrast, the as-synthesized MNP without BSA-coating showed a slight increase in hydrodynamic diameter already at the lowest saline concentration (*c*(NaCl) = 0.05 mol/L) and rapid aggregation at higher saline concentration (*c*(NaCl) = 0.10 and 0.15 mol/L) independent of incubation time. As shown in Figure 7, visual inspection by eye after one-week saline incubation revealed strong precipitation of the as-synthesized MNP, while the BSA-coated MNP appeared as a stable colloidal suspension.

**Figure 8:**
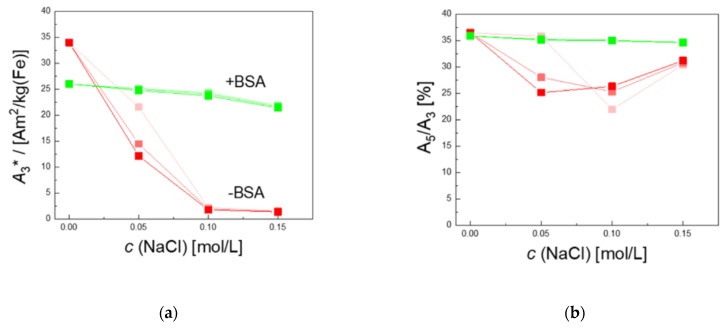
Colloidal stability of MNP in NaCl solution after different incubation times as measured by MPS. MPS parameter *A*_3_* (**a**) of uncoated (red symbols) and BSA-coated MNP (green symbols) at different NaCl concentrations after 2 h, 1 day, and 1 week of incubation (symbols from bright to dark with increasing time). Nearly no changes (less than 10%) of the specific MPS amplitude *A*_3_* were observed in the BSA-coated MNP, indicating colloidal stability up to physiological saline concentrations of 0.15 mol/L. By contrast, the uncoated MNP show a strongly reduced stability visible in the strong *A*_3_* reduction at NaCl concentrations below 0.10 mol/L. As for *A*_3_*, the corresponding parameter *A*_5_/*A*_3_ (**b**) remains unchanged up to physiological NaCl concentration for BSA-coated MNP, while for uncoated MNP, a reduction in the parameter is visible. Since *A*_5_/*A*_3_ is concentration-independent, only a very small fraction (below 5% as estimated from the *A*_3_* drop) of not completely aggregated MNP are contributing to *A*_5_/*A*_3_.

As shown in Figure 8, MPS measurements confirm the results obtained from DCS measurements. The specific MPS amplitude of the MNP without BSA decreases from A_3_* = 33.9 to 24.4 Am^2^/km(Fe) already at the lowest saline concentration *c*(NaCl) = 0.05 mol/L, and almost a complete loss of signal can be observed at higher saline concentrations *c*(NaCl) = 0.10, 0.15 mol/L, independently of the incubation time (see Figure 8a). Modification of MNP by BSA-coating preserved the magnetic signal properties. The A_3_* value remained constant at *c*(NaCl) = 0.05 and 0.10 mol/L and showed a slight decrease from A_3_* =26 Am^2^/kg(Fe) to 22.4 Am^2^/kg(Fe) at *c*(NaCl) = 0.15 mol/L. The *A*_5_*/A*_3_ ratio shown in Figure 8b of BSA-coated MNP remained constant at a high level, exceeding 35% for all saline concentrations independent of incubation time. These high values indicate the high spatial resolution of the MNP and hence a high image quality in MPI. The uncoated MNP showed a significant reduction of the ratio *A*_5_*/A*_3_ for the lower saline concentrations, slightly increasing again at *c*(NaCl) = 0.15 mol/L. Notably, *A*_5_*/A*_3_ is independent of the number of MNP in the sample, and due to the very low *A*_3_* values of the uncoated MNP in a saline environment, this behavior is attributed to the magnetic response of a very small fraction of MNP (below 5% as estimated from the *A*_3_* drop) that are not completely aggregated or agglomerated and emphasizes the high sensitivity of MPS. Based on these promising results, an investigation of the behavior of BSA-coated MNP in different other physiological media such as plasma, full blood, or even living cells will follow soon.

With their physicochemical and magnetic properties, continuously synthesized BSA-coated MNP in aqueous phase offer a superior alternative as tracers for MRI and MPI compared to MNP prepared by other synthetic routes. Small and ultrasmall MNP are suboptimal candidates for MPI. Increasing the core size often leads to increasing magnetic particle–particle interactions with resulting aggregation. Hence, stabilizing the MNP with a suitable coating is crucial to shield these interactions. Choosing the coating material is significant as well. Here, not only the steric stabilization but also the behavior in the physiological environment has to be considered. Pegylation, the attachment of polyethylene glycol (PEG) on the particle surface, is common practice to improve the colloidal stability of nanoparticles in biomedical applications. Kandahar et al. investigated the functionalization of 25 nm single-core MNP with PEG in a time-consuming multi-step process after phase transfer to enhance blood circulation [42]. However, it is frequently reported that PEG can lead to pseudo allergic reactions in clinical applications and can provoke IgE-mediated reactions and recurrent anaphylaxis [65,66]. Chanan-Khan et al. found a direct relation between complement activation and immediate hypersensitivity reactions after initial Doxil^®^ (PEGylated liposomal doxorubicin) dose [67]. Albumin is a safe and very efficient coating approach displaying generally high tolerability. It provides MNP protection from agglomeration mediated by blood constituents such as blood cells, proteins, and high salt concentrations. At the same time, protein coating on the MNP surface can prevent harmful effects of reactive oxygen species [38]. Moreover, the capability to add specific ligands to the functional amino or carboxy groups of the protein empowers BSA-coated MNP for further diagnostic and therapeutic medical applications.

## 3. Materials and Methods

### 3.1. Micromixer Synthesis

Iron oxide nanoparticles were produced continuously using micromixer set-up by precipitation of alkaline solutions of iron chloride in an aqueous medium, as previously reported in [32]. Briefly, solutions of iron chloride, sodium nitrate, and sodium hydroxide (all reagents were used without further purification, purity grade 98%, Sigma Aldrich, Darmstadt, Germany) were mixed in a caterpillar micromixer (Fraunhofer IMM, Mainz, Germany) with symmetric liquid ratios and piped in a 55 °C reaction loop with a residence time of 4 min. Tannic acid (1.7 kDa, Fluka, Schwerte, Germany) was added as a stabilizing agent. MNP were purified by removing unreacted educts and accessing the stabilizing agent via magnetic separation.

Additional centrifugation of 50 mL of MNP dispersion filled falcon tubes at 3300 revolutions per minute (g-force = 2118 relative centrifugal force) for 15 min was used to remove aggregates and superstructure. The pellet was discarded, and the supernatant was taken for further surface modification. The iron concentration *c*(Fe) of the sample was determined photospectroscopically using the phenanthroline protocol (see Section 3.3 below).

### 3.2. Bovine Serum Albumin Coating (BSA-Coating)

Finally, MNP were modified with a protein coating through incubation with 10 mg/mL BSA (Sigma Aldrich, Darmstadt, Germany) in a 50 mM, Ph ≈ 8.5, sodium bicarbonate buffer (Sigma Aldrich, Darmstadt, Germany) at 60 °C for 12 h. The sample was purified by magnetic separation with a LS-column (Miltenyi Biotec, Bergisch-Gladbach, Germany). After passing through the column, the BSA-coated MNP were washed five times with 3 mL sodium bicarbonate buffer to remove excess BSA in the sample and eluted in a 3 mL sodium bicarbonate buffer.

To assess the colloidal stability, NaCl solution at three different saline concentrations (*c*(NaCl) = 0.05, 0.10, and 0.15 mol/L) was added to the centrifuged and BSA-coated MNP, and the resulting changes in hydrodynamic diameter were analyzed by DCS and magnetically by MPS measurements.

### 3.3. Physicochemical Characterization

#### 3.3.1. Transmission Electron Microscopy (TEM)

Core size and shape were obtained by TEM measurements. Samples were prepared by placing a drop of the sample on a carbon-coated copper grid and left at room temperature to evaporate after applying a magnetic field for a short period of time (10 min) to accumulate the MNP. A Zeiss Libra 120 electron microscope (Zeiss, Oberkochen, Germany) at 120 kV acceleration voltage was used to perform the measurement. The images were taken by a CCD camera. The obtained images were evaluated using the open-source software ImageJ (National Institutes of Health, Bethesda, MD, USA) to calculate the mean diameter and standard deviation of the individual nanoparticles (*N* > 10,000).

#### 3.3.2. Differential Centrifugal Sedimentation (DCS)

DCS (or analytical ultracentrifugation) offers detailed information on the dispersion properties of MNP in colloidal systems. In a DCS measurement, sedimentation properties of the particle are measured according to the hydrodynamic particle size distribution [68]; the different size fractions can be differently accelerated in a gravitational field, leading to better fractionalization. Dynamic light scattering (DLS), the standard method to determine the hydrodynamic diameter, gave no results, since the absorption of the nanoparticles with the relatively large core and only a thin tannic acid coating layer was too strong (in contrast to other polymeric coatings such as PEG or dextran that scatter the excitation light well). Thus, no reliable correlation of the fluctuation of the particles could be detected, and size determination with light scattering was not reliably feasible. However, analytical centrifugation is a well-established method to determine size and size distributions, particularly for samples with larger densities such as iron oxide, and also to sensitively display different fractions of polymodal samples. Hence, agglomerated and single-core particles will be accurately detected compared to DLS. Since the density influences MNP sedimentation, a mean density of core (magnetite) and shell material (BSA-coating) of the MNP was taken into account to determine the hydrodynamic diameter by DCS. For BSA, we used a BSA density of *ρ =* 1.41 g/cm^3^ [69] and a hydrodynamic radius of *d_h_*_,BSA_ = 4 nm [63,64]. Assuming a monolayer BSA-coating of 8 nm thickness, an MNP core size of *d*_c_ = 28 nm, and a magnetite density *ρ*_Fe3O4_ = 5.2 g/cm^3^, a mean density *ρ*_BSA-MNP_ = 2.45 g/cm^3^ for BSA-coated MNP was calculated. DCS measurements (CPS Instruments Inc. Measurements, Darmstadt, Germany) were carried out at 20,000 rpm (=21,504 relative centrifugal force) after calibration with a silicon dioxide (SiO_2_) standard (255 nm). Sucrose gradient was built up using 24% to 8% sucrose. Peak maximum and full width at half maximum (FWHM) were evaluated using Origin^®^ software (ADDITIVE Soft- und Hardware für Technik und Wissenschaft GmbH, Friedrichsdorf, Germany).

#### 3.3.3. Zeta Potential Measurements

Zeta potentials in aqueous dispersion were derived from electrophoretic mobility measurements using a Litesizer 500 (Anton Paar GmbH, Ostfildern, Germany). The measurements were carried out in an Anton Paar Omega cuvette at a temperature of 20 °C (temperature equilibration time 30 s). Automatic mode was used for conducting the measurements (automatically chosen parameters: runs processed: 100, adjusted voltage: 200 V). For data evaluation, the Smoluchowski approximation (Henry factor: 1.5) was chosen.

#### 3.3.4. Agarose Gel Electrophoresis

Agarose gel electrophoresis was used to investigate the presence of protein on the surface of the MNP; 1% agarose gel (Carl Roth, Karlsruhe, Germany) was prepared by melting 1 g in 100 mL of 10 mM borat buffer (Sigma Aldrich, Darmstadt, Germany) (pH = 8.3). The mixture was heated using a microwave in intervals of 30 s until the agarose completely dissolved. The gel was poured into a mold with a comb and left for two hours to cool down to room temperature. The agarose gel was placed in an electrophoresis apparatus and a buffer was added to cover the gel. A total of 20 µL of the samples was mixed with 5 µL OrangeG dye (Carl Roth, Karlsruhe, Germany). A total of 20 µL of the mixture was then added to the gel. The power supply of the apparatus was turned on at 290 V and ran for 30 min.

#### 3.3.5. Photospectroscopical Determination of Iron Concentration *c*(Fe)

The iron concentration *c*(Fe) of the nanoparticle samples was determined photospectroscopically using the phenanthroline protocol [70]. A total of 10 µL of nanoparticles was dissolved in 20 µL hydrochloric acid (37%). After complete dissolution, 470 µL of H_2_O was added. A total of 100 µL hydroxylamine hydrochloride (10%) and 700 µL 1,10-phenanthrolinehydrochloride (0.1%) were added to 200 µL of this solution. After a reaction time of 15 min, the absorbance of the formed ferroin complexes was measured by UV-Vis spectrometer (Cary 50, Varian, Palo Alto, CA, USA) at a wavelength of 510 nm, and the iron concentrations were calculated using an iron standard calibration curve (Iron (II,III) Oxid (Sigma Aldrich, Darmstadt, Germany) as standard in the range *c*(Fe) = 1.25 to 40 mM).

### 3.4. Magnetic Characterization

#### 3.4.1. Magnetic Particle Spectroscopy (MPS)

MPS measurements of single nanoparticle samples were performed using a commercial magnetic particle spectrometer (MPS-3, Bruker, Ettlingen, Germany) operating at an amplitude *B*_ex_ = 25 mT and a frequency *f*_0_ = 25 kHz. MPS detects the non-linear dynamic magnetic response of MNP exposed to an alternating magnetic field from which their MPI performance can be assessed. Originally, MPS was developed to assess the performance of MNP tracer materials in MPI. However, here, we use MPS to quantitatively reveal changes in the dynamic magnetic behavior of MNP mediated by the direct environment of the MNP, e.g., as a consequence of surface modification by BSA-coating or physiological conditions as different saline concentrations.

For MPS measurement, a fast reaction tube (Applied Biosystems^®^, MicroAmp, Thermo Fisher Scientific, Schwerte, Germany) containing a sample volume of 30 µL was placed in the detection coil of the MPS system. The induced magnetization could be measured simultaneously by the coils. By Fourier transform of the detected time signal, the spectral components of an MPS measurement were obtained, showing distinctive amplitudes at odd multiples (harmonics) of the excitation frequency *f*_0_. We used two characteristic parameters of the MPS harmonic spectra, the amplitude of the third harmonic normalized to the iron amount of the sample, *A*_3_*, and the concentration-independent ratio between the 5th and 3rd harmonic, *A*_5_/*A*_3_. Both values are correlated to the MPI performance, with the general observation that the higher the *A*_3_* and *A*_5_/*A*_3_, the better the MPI images. The specific signal amplitude A_3_* indicates the sensitivity of the MNP to give a dynamic magnetization response at the chosen excitation frequency *f*_0_ per unit amount of iron, and the ratio *A*_5_*/A*_3_ is used to describe the shape of the harmonic spectrum to assess the resolution capacity.

Two commercial MNP systems were considered as references with high MPS performance. For the MRI liver contrast agent Resovist (Meito Sanyo, Japan), we used the literature values *A*_3_* = 8.7 Am^2^/kg(Fe) and *A*_5_/*A*_3_ = 38.4% [71]. Furthermore, carboxyl iron oxide MNP with 30 nm core diameter (SHP-30-10, Ocean NanoTech, US) measured by MPS at *B*_ex_ 25 mT resulted in A_3_* = 32.8 Am^2^/kg(Fe) and A_5_/A^3^ = 22.2%.

To analyze the colloidal stability of MNP at different saline concentrations by MPS, 10 µL aliquots of as-synthesized, centrifuged, and BSA-coated MNP incubated with a final concentration of c (NaCl) = 0.05, 0.10, and 0.15 mol/L were taken after 2 h, 1 day, and 1 week. The MPS measurements were carried out at *B*_ex_ = 25 mT and *T* = 37 °C.

#### 3.4.2. NMR Relaxivities *r*_1_ and *r*_2_

MRI imaging properties were investigated by measuring longitudinal *T*_1_ and transversal *T*_2_ proton relaxation times for an MNP sample of 200 μL volume diluted to different iron concentrations c (Fe). The relaxation time measurements were carried out on a Minispec mq60 relaxometer (Bruker, Ettlingen, Germany) at *T* = 37 °C and a magnetic field of 1.5 T (60 MHz proton resoncane frequency). For *T*_1_, a 2-pulse inversion-recovery sequence with a fixed relaxation delay of at least 5 *T*_1_ was used, *T*_2_ was determined employing a Carr–Purcell–Meiboom–Gill sequence, which consists of a 90° pulse followed by a series of 180° pulses, ideally covering the full decay of the signal.

From the graphs of the iron-concentration *c*(Fe)-dependent relaxation times *R*_1_ = 1/*T*_1_ and *R*_2_ = 1/*T*_2_, the corresponding relaxivities *r*_1_ and *r*_2_ (in units of L·mmol^−1^·s^−1^) were determined:

(1) $$\frac{1}{T_{i}}=\frac{1}{T_{i,H_{2}O}}+r_{i}\;c\left(\mathrm{Fe}\right),\;\;\;\;\;\;i=1,2$$

For graphical presentation, the measured relaxation rates of pure water samples have been subtracted (*R*_1,H2O_ = 0.25952 s^−1^ and *R*_2,H2O_ = 0.29 s^−1^).

#### 3.4.3. Linear Dynamic Susceptibility Measurements (ACS)

Room temperature (*T* = _295_ K) linear magnetic AC susceptibility (ACS) of MNP was measured with a commercial AC susceptometer (DynoMag, RISE Acreo, Gothenburg, Sweden). For the measurements, a sample volume of _200_ µL MNP suspension was filled into a quartz glass cuvette, and the real *χ′*(*f*) and imaginary *χ*′′(f) magnetic susceptibility were acquired in the frequency range _1_ Hz to _100_ kHz at an excitation amplitude of _0_._2_ mT. The initial mass susceptibility *χ*_0_ (in units m_3_/kg(Fe), normalized to the sample iron mass) was obtained by extrapolation of the real part susceptibility *χ′*(*f*)|*^f^*→0.

## 4. Conclusions

Surface modification was performed to optimize the stability and performance of continuously manufactured MNP for molecular imaging applications such as MPI and MRI in a physiological environment. Protein coating of electrostatically stabilized nanoparticles with BSA led to a significant improvement in dispersion stability in the presence of an increasing concentration of NaCl solution. No drop in signal amplitude and constant *A*_5_*/A*_3_ values prove the good performance of the MNP as MPI contract agents. Additionally, *r_1_* and *r_2_* values are promising to apply MNP as MRI contrast agents. Since Resovist was withdrawn from the market, our MNP are promising candidates to further accelerate the research on MPI and MRI molecular imaging.

Even after 40 years of MRI contrast agent developments for clinical use (the first MRI contrast agent, introduced in 1981, was ferric chloride), newer and safer MRI agents capable of specifically targeting organs, sites of inflammation, and tumors are still demanded. The presented micromixer technique to synthesize MNP-based contrast agents with tailored magnetic properties provides a major contribution to future developments.

The same applies to MPI being under development for medical imaging of the cardiovascular system, for oncology or for stem cell tracking, where the structural, magnetic, and surface properties of the MNP are of utmost importance and need further optimization. With its excellent control of particle size, crystal growth, and scalability, the micromixer technique in combination with additional surface modification can also foster the further development of high-performing tracer materials for this promising imaging modality.

## Figures and Tables

**Table 1:** Properties of the investigated MNP systems together with the two commercial MNP systems, Resovist and SHP-30 MNP. System information and coating are provided in the first two columns, followed by magnetic parameters obtained by MPS (amplitude of the 3 rd harmonic normalized to iron amount, A3*), ACS (initial susceptibility *χ*_0_), and NMR (relaxivities *r*_1_ and *r*_2_ and the ratio *r*_2_/*r*_1_ measured at 1.5 T).

Sample System	Coating	A_3_* Am^2^/kg(Fe)	*χ_0_* m³/kg(Fe)	*r*_1_ L/(mmol·s)	*r*_2_ L/(mmol·s)	*r*_1_/*r*_2_
as-synthesized MNP single core, *d*_c_ = 27.7 nm, cont. micromixer synthesis	tannic acid	21.3	0.062	11.6(9)	482(8)	42
centrifuged MNP	tannic acid	33.9	0.084	13.6(6)	620(7)	46
BSA-coated	bovine serum albumin	26	0.072	6.2(4)	600(10)	97
Resovist multi-core ^2^, 6 nm cores bimodal size distribution mean cluster size 24 nm	carboxydextran T1.8 kDa	8.7	-	7.4 ^1^ 8.7 ^2^	95 ^1^ 61 ^2^	15
SHP-30 single core, thermal decomposition, 30 nm +/− 2.5 nm	amphiphilic coating with carboxylic acid groups	32.8	-	8.0(5)	660(10)	83

^1^ Literature values for Resovist relaxivities at 1.5 T: *r*_1_ = 7.4 L·mol^−1^·s^−1^ and *r*_2_ = 95 [1], or *r*_1_ = 8.7 L·mol^−1^·s^−1^ and *r*_2_ = 61 L·mol^−1^·s^−1^ [62]. ^2^ Size distribution values for Resovist from [1,29]. Note that the uncertainty of a *r*_1_ or *r*_2_ value is denoted in brackets, e.g., 11.6(9) L·mol^−1^·s^−1^ is short-hand notation for 11.6 ± 0.9 L·mol^−1^·s^−1^.

## Data Availability

Data is contained within the article.
